# Molecular Characterization of HEV Genotype 3 in Italy at Human/Animal Interface

**DOI:** 10.3389/fmicb.2020.00137

**Published:** 2020-02-05

**Authors:** Luca De Sabato, Ilaria Di Bartolo, Daniele Lapa, Maria Rosaria Capobianchi, Anna Rosa Garbuglia

**Affiliations:** ^1^Department of Food Safety, Nutrition and Veterinary Public Health, Istituto Superiore di Sanità, Rome, Italy; ^2^Laboratory of Virology, “L. Spallanzani” National Institute for Infectious Diseases, IRCCS, Rome, Italy

**Keywords:** hepatitis E virus, hepatitis E, subtype, prevalence, zoonosis, Italy

## Abstract

Hepatitis E virus (HEV) is an emerging public health issue in industrialized countries. In the last decade the number of autochthonous human infections has increased in Europe. Genotype 3 (HEV-3) is typically zoonotic, being foodborne the main route of transmission to humans, and is the most frequently detected in Europe in both humans and animals (mainly pigs and wild boars). In Italy, the first autochthonous human case was reported in 1999; since then, HEV-3 has been widely detected in both humans and animals. Despite the zoonotic characteristic of HEV-3 is well established, the correlation between animal and human strains has been poorly investigated in Italy. In the present study, we compared the subtype distribution of HEV-3 in humans and animals (swine and wild boar) in the period 2000–2018 from Italy. The dataset for this analysis included a total of 96 Italian ORF2 sequences (300 nt long), including both NCBI database-derived (*n* = 64) and recent sequences (2016–2018, *n* = 32) obtained in this study. The results show that subtype 3f is the most frequent in humans and pigs, followed by the HEV-3e, HEV-3c and other unassignable HEV-3 strains. Diversely, in wild boar a wider group of HEV-3 subtypes have been detected, including HEV-3a, which has also been detected for the first time in a human patient in Central Italy in 2017, and a wide group of unassignable HEV-3 strains. The phylogenetic analysis including, besides Italian strains, also sequences from other countries retrieved from the NCBI database, indicated that human Italian sequences, in particular those of HEV-3f and HEV-3e, form significant clusters mainly with sequences of animal origin from the same country. Nevertheless, for HEV-3c, rarely detected in Italian pigs, human sequences from Italy are more correlated to human sequences from other European countries. Furthermore, clusters of near-identical human strains identified in a short time interval in Lazio Region (Central Italy) can be recognized in the phylogenetic tree, suggesting that multiple infections originating from a common source have occurred, and confirming the importance of sequencing support to HEV surveillance.

## Introduction

Hepatitis E virus (HEV) is a quasi-enveloped RNA virus with a single stranded genome positive-sense of approximately 7.2 kb. It belongs to the *Hepeviridae* family, genus *Orthohepevirus* which includes 4 species *(Orthohepevirus A-D).* Species *A* is divided into 8 genotypes, of which genotype 1 and 2 (HEV-1 and HEV-2) infect only humans, HEV-5, HEV-6, and HEV-8 are only detected in animals (Forni et al., 2018) and HEV-3, HEV-4 and HEV-7 (Lee et al., 2016) are zoonotic. HEV-1 and HEV-2 have an intra-human cycle and cause large epidemics due to poor sanitary conditions in developing countries, while in Europe and the US HEV-3 is the most common; HEV-4 is mostly diffused in Asia. Both HEV-3 and HEV-4 are zoonotic and the main animal reservoirs are swine, wild boar and deer (Primadharsini et al., 2019).

In Europe, HEV-3 is considered an emerging foodborne pathogen; the number of patients with hepatitis E has been increasing in the last 10 years probably because of higher clinicians’ awareness coupled to increased circulation of the virus (Kamar et al., 2012, 2017; Domanovic et al., 2017). In Europe, although HEV-3 is the most common in both humans and animals, cases of HEV-4 have been reported (Colson et al., 2012; Garbuglia et al., 2013) but in pigs this genotype has been detected sporadically (Monne et al., 2015). The transmission is mainly caused by consumption of undercooked or raw contaminated food of animal origin (pig, deer, and wild boar meat). In humans, hepatitis E is an acute hepatitis, usually self-limiting. In immunocompromised patients extra-hepatic manifestations and chronic infection have been described, but only HEV-3 may cause persistent infections (Kamar et al., 2015).

In Europe, the reported seroprevalence in the general population or in blood donors is highly variable, ranging between 6.1% and 52.5% (Mansuy et al., 2011; Capai et al., 2019); some hyperendemic areas with high seroprevalence have been described (Müller and Koch, 2015; Zaaijer, 2015; Adlhoch et al., 2016; Mansuy et al., 2016; Bura et al., 2017). The different seroprevalence values reported in different countries or regions of the same country may partially depend on different assays used (Norder et al., 2016; Sommerkorn et al., 2017) or on dietary habits such as consumption of raw meat (Slot et al., 2017) or raw dried pig liver sausages (Lucarelli et al., 2016). Furthermore, the seroprevalence observed in several European countries is higher than expected on the basis of reported cases, suggesting HEV underdiagnosis and/or asymptomatic infections (Ricci et al., 2017). In Italy, a mean seroprevalence of 8.7% has been observed in blood donors. Some Italian regions are considered hyperendemic, i.e., 10.0 to 15.0% seroprevalence is reported in Lazio, Umbria, and Marche and >22.0% in Abruzzo and in Sardinia (Spada et al., 2018); in the Lazio region, a retrospective study performed on people who received counseling and underwent serological tests for anti-HIV antibody between 2002 and 2011 showed an overall anti-HEV IgG prevalence of 5.38%, with a variation over time fluctuating within a 3-year period, and an increase of 4.0% per year of participants’ age (Lanini et al., 2015). HEV-3 is the most frequently detected in humans, pigs and wild boar In Italy. The reported risk factors among confirmed hepatitis E cases are the consumption of undercooked pork meat and wild boar sausages (La Rosa et al., 2011). As a matter of fact, sequence-based direct evidence of foodborne transmission has been recently provided in an Italian citizen who acquired a HEV-3i strain from figatelli (pork liver sausage) bought in France (Garbuglia et al., 2015).

To date, only one serotype has been described and the only way to determine the source of infection or to trace back the contaminated food is based on sequence analysis (Tei et al., 2003; Takahashi et al., 2004; Colson et al., 2010; Dalton et al., 2011). HEV strains belonging to the same genotype are further classified in subtypes or genetic variants based on sequence p-distance among strains. The HEV-3 strains are classified in 12 subtypes (HEV-3a to HEV-3l) differently distributed worldwide. The HEV-3c, HEV-3e and HEV-3f are the most common in Europe (Lu et al., 2006; Lapa et al., 2015). In addition, several unassigned subtypes and uncommon strains have been recently proposed (Smith et al., 2016). HEV-3c, HEV-3e, and HEV-3f have been reported not only in humans and animals (pigs and wild boar) but also in contaminated food items of pork (pig liver sausage) and wild boar (sausages) (Colson et al., 2010; Di Bartolo et al., 2015; Garbuglia et al., 2015; Montone et al., 2019) and in the environment (Di Profio et al., 2019).

Changes over time in the subtype circulation have been observed in Europe. Subtype HEV-3f represented 90.0% of human infections in South Western France in the period 2003–2005, and the incidence of this subtype dropped to 65.0% during 2012-2014 due to the increase of subtype HEV-3c circulation (Lhomme et al., 2015; Nicot et al., 2018). In England, HEV-3e, HEV-3f, and HEV-3g predominated before 2009, while HEV-3c, which first appeared in 2008, become the predominant variant in 2012 (Ijaz et al., 2014; Harvala et al., 2019).

In Italy, only few data are available on HEV subtype circulation in humans. Few studies described the detection of HEV-3 strains in human cases (La Rosa et al., 2011; Romano et al., 2011; Festa et al., 2014; Garbuglia et al., 2015; Lucarelli et al., 2016; Alfonsi et al., 2018; Marrone et al., 2019) and their classification into the HEV-3c and HEV-3e subtype (Festa et al., 2014; Garbuglia et al., 2015).

Despite the increasing number of papers reporting HEV-3 detection in humans in Italy, the relationship among humans, swine and wild boar strains has not been extensively addressed (Montesano et al., 2016). The aim of the present study was to merge the sequence information of strains circulating in Italy in animals (swine and wild boar) and humans, to evidence possible genetic correlation and eventually trace the HEV origin of human strains circulating in Italy.

## Materials and Methods

### Constitution of Sequence Dataset

#### Dataset of Italian Sequences

All HEV-3 sequences available in NCBI database (as of June 2019) were screened, and, for the purpose of the first part of study, all those of Italian origin (humans, pigs, and wild boar), overlapping to a 300 bp of ORF2, corresponding to nucleotide positions 5988–6287 nt of HEV complete genome Acc. n° NC_001434.1, were selected and included in the alignment. Sequences identical to each other and/or shorter than 300bp were discarded. The genomic region of the ORF2 (300 bp) was selected because it is the most represented in the NCBI (Ricci et al., 2017) and overlaps with the genome fragment of the ORF2 sequenced in this study. The final dataset of Italian sequences retrieved from NCBI included 22 swine, 32 wild boar and 10 human strains collected from years 2000 (first swine strain described) to 2018. Since no recent HEV-3 Italian ORF2 sequences were available in the NCBI database, the initial dataset was integrated with 32 novel HEV-3 sequences not described before: *n* = 23 of human origin, obtained in the years 2016–2018, and *n* = 7 obtained in previous years, 2011–2015; 2 recent sequences (collected in 2018) of swine origin were included as well.

#### Dataset of Italian and Worldwide Sequences

Subsequently, Italian HEV-3 sequences were compared to those from pigs, wild boars and humans available in the database worldwide, using the NCBI BLASTn^1^. MEGA7 software^2^ was used for the alignment. Reference HEV-3 sequences of established and recently proposed subtypes (*n* = 15) (Smith et al., 2016; Miura et al., 2017; De Sabato et al., 2018) and 130 sequences showing closest relatedness (≥93.0% nt. identity) to the novel Italian sequences were included in phylogenetic analysis, for a total of 145 sequences included in the analysis (Table 1).

**TABLE 1:** Dataset description of sequences of human and animal origin detected worldwide and used to build the tree.

	EU			Non-EU				Total
Subtype	Hu	Sw	Wb	Hu	Mon	Sw	Wb	
3*	1	1	1			2		5
3a	2	2		3		1		8
3b				1		1	1	3
3c	45	8	1	1				55
3e	6	3	1	2	1	4		17
3f	32	12		8		2		54
3g						1		1
3h	1							1
3i			1					1
3j						1		1
3k				2		1		3
3l	1	1						2
Total	88	27	4	17	1	13	1	151**

*, Not assignable to any subtype; **, 15 reference strains; Hu, humans; Sw, pig; Wb, wild boar; Mon, monkey; EU, European strains; Non-EU, non-European strains.

### Human and Animal Samples From Which the Novel HEV-3 Sequences Used in This Study Were Obtained

Following the designation of the Laboratory of Virology of the National Institute for Infectious Diseases “L Spallanzani” (INMI) as Regional Reference Center for HAV and HEV in late 2015, all diagnostic samples with HEV IgM were analyzed for the presence of HEV genomes and those resulting HEV-RNA positive were sequenced. In the period January 2016 to December 2018, 161 serum samples from anti-HEV IgM-positive patients were analyzed, and sequences were obtained from 44 patients, yielding 23 HEV-3 infections. Only randomly selected samples had been sequenced in previous years (7 HEV-3 strains). The study was approved by the INMI Ethical Committee, and the analysis performed after patient anonymization.

In 2018, 15 pool fecal samples were collected from pen floor in 2 pig farms housing weaners and tested for the presence of HEV RNA; HEV-3 sequences were obtained from both farms.

### Laboratory Methods

Hepatitis E virus infection was diagnosed by detecting IgG/IgM anti-HEV antibodies using a commercial enzyme-linked immunosorbent assay (DIA.PRO, Milan, Italy), according to the manufacturer’s instructions. For molecular analysis, nucleic acids were extracted with QIASYMPHONY automated instrument (QIAGEN, Hilden, Germany). Internal RNA control template QuantiFast (QIAGEN, Hilden, Germany) was added prior to lysis step and viral RNA extraction to monitor the presence of inhibitors and to check nucleic acid extraction efficiency by performing a quantitative Real-time with QuantiFast Pathogen RT-PCR + IC Kit (QIAGEN, Hilden, Germany). RNA obtained was reverse transcribed and amplified by One-STEP RT-PCR (QIAGEN, Hilden, Germany) following the manufacturer’s recommendations. The amplification target was a 412 bp fragment within the ORF2 (positions 5953–6363 respect to the E116-YKH98C strain, AB369687) (Mizuo et al., 2002). Briefly, first round was performed using a PCR protocol with reverse transcription at 50°C for 30 min followed by a denaturation step of 15 min at 95°C, and subsequent 35 cycle at 94°C for 1 min, at 55°C for 30 s and 72°C for 1 min, followed by a final extension at 72°C for 7 min. Nested PCR was performed using TaqGold DNA polymerase (Applied Biosystem Forster, CA, United States) at 94°C for 15 min followed by 35 cycles at 94°C for 30 s, 56°C for 30 s and 72°C for 45 s.

RT-PCR products with expected size were purified using a QIAQUICK PCR products kit (Qiagen, Hilden, Germany); the PCR amplicons were sequenced with the second PCR round primer set, using the method of BigDye terminators, on the 3500 XL sequencing instrument (Applied Biosystem Forster, CA, United States). Two sequences were obtained from pigs (Acc. n° MN546866; MK689362) following the same protocols described above for humans. Table 2 reports the accession numbers of the novel human sequences from Lazio Region (Central Italy) used in this study.

**TABLE 2:** Sequence information of human HEV-3 sequences from Lazio region.

Sequence ID_Collection date	GenBank acc. number	Subtype
2120_2011	MN509469	3c
2122_2011	MN509470	3e
1203_2012	MN509471	3f
1205_2012	MN444846	3f
1313_2013	MN444845	3f
1402_2014	MN444847	3e
1516_2015	MN444844	3f
1602_2016	MN444839	3c
1603_2016	MN444842	3f
1604_2016	MN444838	3e
1609_2016	MN444840	3f
1610_2016	MN444843	3f
1611_2016	MN444841	3e
1706_2017	MN432489	3f
1707_2017	MN444828	3f
1708_2017	MN444829	3f
1712_2017	MN444830	3f
1714_2017	MN444831	3f
1715_2017	MN444832	3f
1718_2017	MN444833	3f
1719_2017	MN444834	3f
1725_2017	MN444835	3f
1728_2017	MN444836	3e
1736_2017	MN444837	3a
1809_2018	MN444853	3f
1813_2018	MN444852	3f
1814_2018	MN444848	3c
1820_2018	MN444852	3f
1823_2018	MN444849	3f
1825_2018	MN444850	3f

### Phylogenetic Analysis

The maximum likelihood (ML) phylogenetic tree was constructed with the Tamura–Nei parameter model as suggested by the MEGA 7 software model test based on 1,000 bootstrap replications. Reference HEV-3 sequences of established and recently proposed subtypes (*n* = 15) (Smith et al., 2016; Miura et al., 2017; De Sabato et al., 2018) were included in the tree for the HEV-3 assignment. Those sequences not belonging to any subtypes defined so far were aligned with sequences of the HEVnet dataset (Mulder et al., 2019) using the public HEVnet typing tool^3^.

## Results

### Subtype Distribution of Human and Animal Italian HEV-3 Strains

The first Italian HEV-3 sequences suitable for the present analysis dated back to 2000 for pigs, 2003 for humans and 2012 for wild boars.

Even though the time interval covered by the study sequences was not overlapping for the 3 HEV hosts, the overall subtype distribution is reported in Table 3.

**TABLE 3:** Subtypes HEV-3 distribution of animal and human strains detected in Italy (this study and downloaded at the NCBI database; collected from 2000 to 2018).

Subtype	Human**	Swine	Wild boar
3a	1	0	2
3c	6	1	6
3e	7	7	0
3f	24	11	8
3l	1	2	0
Unclassified*	1	3	16
Total	40	24	32

**First human strain in Italy detected in 2003. *Not assignable to any subtype.

As can be seen, subtype assignment was achieved in 75 over 96 sequences, wild boar sequences being the most frequent in the unassigned group. Among the successfully subtyped strains, subtype HEV-3f was predominant in all species, followed by HEV-3e in all but wild boar species; HEV-3a and HEV-3l were scarcely represented in all species, while HEV-3c seemed to be slightly more frequent in wild boar (18.7%) than in humans (15.0%), and rare in pigs (<5.0%) (Table 3). The three swine (KJ508211, KF888265, and KF939862) and the wild boar (MH836530) sequences were not assigned to any subtypes either using the list of references strains (Smith et al., 2016) or by HEVnet typing tools. Differently, the three wild boar clusters including unassigned sequences, each one represented by MF959765, MK390970, and MF959764 were assigned to three provisional novel subtypes by the HEVnet typing tools (named in the typing tools: 3u(p), 3w(p), and 3t(p), respectively). Since for the three clusters only one full genome was available, the Italian strains for which a full genome sequences are available would represent the reference strains of the putative novel subtypes.

### Phylogenetic Relationships Between Human and Animal HEV-3 Sequences

**FIGURE 1:**
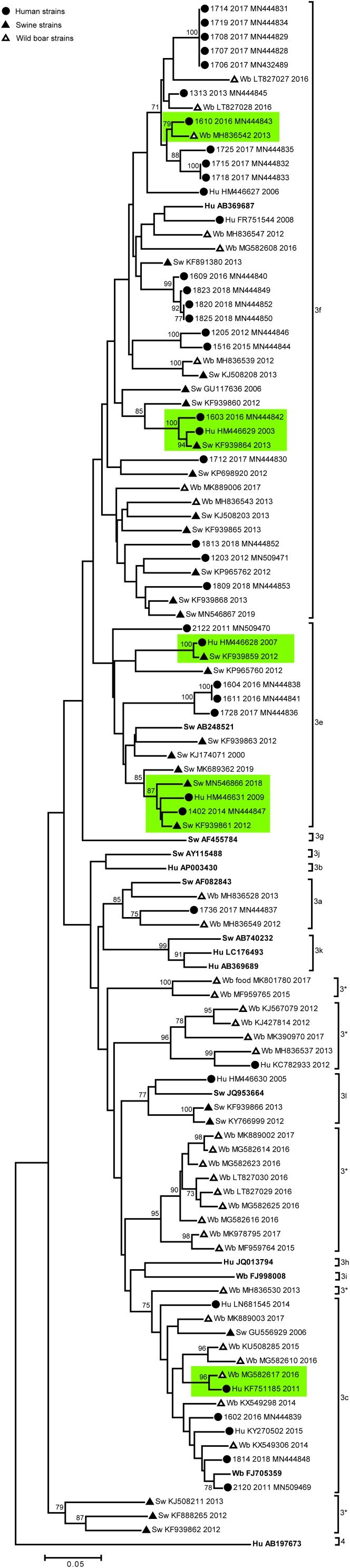
based on a 300 nt fragment of the partial ORF2 region of Italian HEV strains (*n* = 96) of human and animal (swine and wild boar) origin and 13 HEV subtype reference strains. The HEV-4 sequence (Hu AB197763 JP) was used as outgroup. The maximum likelihood tree was produced using the Tamura–Nei parameter model based on 1,000 bootstrap replications and bootstraps values >70 are indicated at their respective nodes. The Italian entries downloaded from NCBI database includes host (Hu, human; Sw, swine; Wb, wild boar) accession number and year of collection. The human Italian strains from this study were reported by sequence name and accession number. The HEV reference strains are reported in bold. Human and animal sequences forming strictly related clusters are highlighted in green. Symbol disclosure is included in the figure.

We next performed phylogenetic analysis of the Italian strains to identify genetic correlations between human and animal sequences. Results are shown in Figure 1. Some Italian human strains detected in this study were identical or strictly related among each other (displaying nucleotide identity >99.0%), in particular 5 strains HEV-3f detected in 2017 (1707-2017; 1708-2017; 1714-2017; 1719-2017; and 1706-2017) formed a statistically supported cluster in the phylogenetic tree (Figure 1), with null (4 strains) or very short p-distance (1 strain, 99.8% nt id) among each other. Similarly, three human strains (HEV-3f) detected in 2018 formed a significant cluster with another human Italian strain detected in 2016 (1609-2016), displaying 99.4% nt id. to each other. Finally, two HEV-3e strains (1604-2016 and 1611-2016) both detected in 2016, were identical.

Among the Italian animal strains, identical sequences were not detected; the closest nucleotide identity was observed between two swine Italian strains detected in 2012 and 2013 and belonging to HEV-3l (99.9% nt.id) (KF939866 and KY766999).

Except for the subtypes HEV-3f, HEV-3c, HEV-3a, wild boar sequences included mostly HEV-3 sequences with unassignable subtype (*n* = 16), showing identical p-distance with different HEV-3 subtypes (Figure 1; indicated with 3^∗^).

A unique significant cluster between animal sequences was observed, including one swine and one wild boar HEV-3f strain (KJ508208 and MH836539).

To address the possible autochthonous zoonotic origin of human infections, we considered clusters including both animal and human sequences (Figure 1). Although, the HEV-3f and HEV-3e subtypes are the most common among both Italian human and swine strains, no identical human and animal strains were detected. However, in the HEV-3f, HEV-3e and HEV-3c subtypes some clusters showed strict sequence correlations (highlighted in green in Figure 1); in particular the human 1603-2016 (HEV-3f) formed a sub-cluster (98.0% nt. id.) with both human (HM446629) and swine (KF939864) sequences described in 2003 and 2016, respectively. Similarly, in the HEV-3e cluster, the human strain 1402-2014 showed a 96.0–98.0% nucleotide identity (nt. id.) with one human (96.9% nt. id.; HM446631 in 2009) and three Italian swine strains: KF939861 (98.0% nt. id.; 2012), MN546866 (96.0% nt. id.; 2018), MK689362 (94.0% nt. id.; 2018). In these sub-clusters, human strains were detected over different years (2003 vs. 2016; 2009 vs. 2014) in different area of the country (northern and central Italy). The highest nucleotide identity (up to 99.0%) among animal and human Italian strains belonging to the HEV-3f, was displayed between human (HM446629, HM446628) and swine (KF939864, KF939859) strains detected in different years (2007 vs. 2012; 2003 vs. 2013), both originating from Northern Italy. Besides the high heterogeneity of Italian wild boar strains, mostly belong to unassignable HEV-3 subtypes, three strains, belonging to HEV-3f (MH836542) and HEV-3c (MG582617) displayed up to 99.0% identity with human strains detected in different years and areas of the country (Figure 1).

**FIGURE 2:**
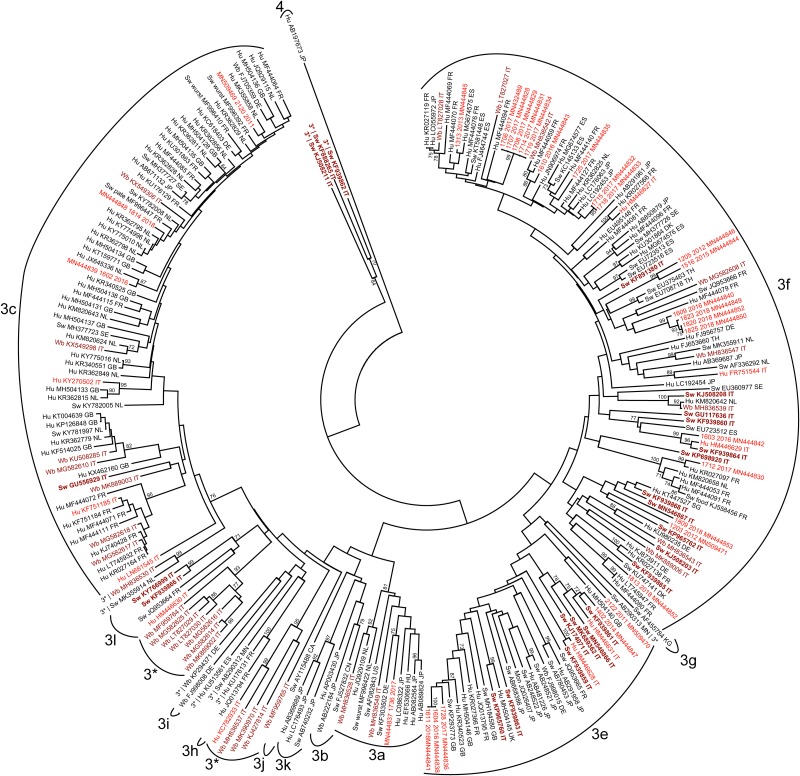
Phylogenetic analysis based on the 300 nt fragment of the partial ORF2 region of HEV-3 strains derived of human or animal origin. A total of 244 HEV-3 sequences have been included, and the HEV-4 sequence (Hu AB197763JP) was used as outgroup. The maximum likelihood tree was produced using the Tamura–Nei parameter model based on 1,000 bootstrap replications and bootstraps values >70 are indicated at their respective nodes. Each entry includes host (Hu, human; Sw, swine; Wb, wild boar; Mon, monkey), accession number and countries origin of strains. The human Italian strains from this study were reported by sequence name and accession number. Italian sequences are highlighted as follows: human sequences are in red, animal sequences (swine and wild boar) are in brown, of which swine sequences are in bold.

In the subsequent step, we included in the phylogenetic analysis all HEV-3 sequences sharing at least 93.0% nt id with the Italian sequences. The results are shown in Figure 2.

Some human strains from Italy, both HEV-3c and HEV-3f, were more strictly correlated to HEV-3 human strains described in Europe than with Italian strains. Among them, the HEV-3c Italian human strains (2120-2011, 1814-2018, 1602-2016, KY270502, KY270502, and KF751185) and only sporadically the HEV-3f (1712 2017, 1813-2018) displayed a nt. id. ranging between 93.0% and 99.0% with numerous sequences available online, mainly detected in humans in Europe, among which the highest genetic correlation (97.0–99.0% nt. Id) was observed with human strains reported in France, Netherlands and in the United Kingdom (MF4444071 FR; KR362815 GB; KY775016 NL; KR362795 NL; and MK355858 NL). Correlation with non-European sequences was only observed for two human Italian HEV-3f sequences (1715-2017 and 1718-2017), sharing 99.0% nt. id. to each other, and related to a non-European human sequence detected in Japan (LC192453).

The human strain 1736-2017 was classified as HEV-3a, 91.0% nt. id. with the prototype strain (AF082843, Meng), but displayed only a limited nucleotide identity (<93.0% nt. id.) with the other HEV-3a strains including the recently detected Italian wild boar strains (MH836549, MH836528), while the closest sequence (93.2% nt. id.) was that of a swine strain from Germany (KF303502). Among Italian animal strains not significant correlation with other non- Italian strains was observed. Nevertheless, within the HEV-3f, swine and wild boar strains (KJ508208 and MH836539) formed a supported and strict cluster with a human strain detected in the Netherland (KM820642) and another Italian swine strains was related to a strain detected in a patient in France (KR027099).

Similarly, the HEV-3c sequences detected in Italian wild boar are related to some human strains detected in non-Italian European countries (Netherland and United Kingdom).

## Discussion

Analyses conducted by sequence alignment and by establishing correlation among strains using phylogenetic analyses confirmed a genetic diversity of Italian HEV-3 strains. Despite the large variation in the number of sequences obtained for each year, the most common subtype was HEV-3f for both human and pig strains. The HEV-3f was predominant (*n* = 24) in humans both before and after 2016 when a systematic sequence collection of IgM HEV-positive sera in central Italy (Lazio Region) was established.

The HEV-3f was also the most commonly identified subtype in humans in Spain, France (Adlhoch et al., 2016) and in Belgium until 2015 (Suin et al., 2019). However, in some European countries a shift of subtypes has been observed among HEV-3 human strains, so that subtype HEV-3c is now predominant in England, Wales, Netherlands, and Germany (Suin et al., 2019). Also in Belgium the HEV-3c has become the most common subtype (since 2016), followed by the HEV-3f and HEV-3e, which are frequently reported in both humans and animal reservoirs (Adlhoch et al., 2016; Suin et al., 2019).

In Italy, this shift in HEV-3 subtype circulation was not observed either in humans or in animals. Among Italian pig sequences available online only one HEV-3c was identified and HEV-3c strains detected in wild boar are more strictly correlated to European human strains than to the Italian ones. This result could be attributed to wide circulation of pigs over European countries and the HEV-3c may have been imported to Italy. In Italy, the live import of piglets market (almost 1.6 million head) is mainly from Denmark and Netherlands (AHDB, Agriculture and Horticulture Development Board)^4^ where the HEV-3c subtype has been described as the predominant subtype (Adlhoch et al., 2016).

Diversely, for the HEV-3f and, to a lesser extent, HEV-3e, human and pig Italian strains are more similar to each other than to European strains. The similarity of Italian human strains (e.g., HEV-3f), when observed, is not restricted to a short time interval in the time lapse considered in this study. No geographical correlation could be established, since most recent human sequences correspond to human cases which occurred in Lazio Region (Central Italy).

The phylogenetic analysis also indicated that human Italian sequences clustered not only with sequences of animal origin circulating in the same territory, but also with human and animal sequences from other countries, suggesting that meat/food from these countries may act as virus carrier. However, it is not easy to trace movement of HEV pig strains, because besides movement of live animals, the import/export of fresh and cured meat is frequent among European countries. However, the Italian pork export is lower than imports (AHDB)^5^. In Italy, which account for the 6.0% production of pig meat in Europe (1.470 thousand tonnes), pork meat is imported mainly from EU countries especially from Germany which is the main pig meat suppliers in Europe^6^. However, cured Italian pork products are also exported. In conclusion, understanding the trade of live pigs and pork would be important to control the HEV spreading. However, the complexity of trades makes impossible to predict strains movement but sequence analyses would help to understand their movement and possibly prevent spreading of emerging strains if arose.

The detection of identical human strains in the same year and frequently in the same month suggests the occurrence of small transmission clusters. This may be linked to a common source of infection but with the limited data available and the short sequence stretch analyzed, a definitive interpretation is difficult. It is noteworthy that only after 2016, when a stricter surveillance of cases supported by sequencing was established in Lazio Region (Central Italy) for HEV, could a suspected outbreak be hypothesized on the basis of sequence data. Most probably the number of sequences available before 2016 lead to a limited coverage to allow the identification of genetically correlated strains.

No identical sequences were detected among human and animal strains. This could be due to difficulties in tracing back the origin of infections because of the movement of animals or for the long incubation period of the infection, but may also be linked to the evolution of the strains in different hosts (Brayne et al., 2017).

In this study, the presence of the HEV-3a strain in a human case is firstly described in Italy. This subtype circulates predominantly in Japan and in the United States. More recently, the HEV-3a has also been described in Europe (Germany, Austria, Croatia, Hungary, and Belgium) (Reuter et al., 2009; Jemersic et al., 2019; Suin et al., 2019), although it can be considered still rare. In Italy, the HEV-3a has been recently described in wild boar (Di Pasquale et al., 2019) but it has never detected in other animal reservoirs. The Italian HEV-3a human and wild boar strains shared a limited nucleotide identity among them and with the other HEV-3a strains reported in Europe. The patients infected by HEV-3a identified in this study is a man who had recently traveled to Albania; he also reported the consumption of raw grocery in Italy (nearby Rome), but, among those who had been potentially exposed in that occasion, he was the only one who contracted HEV infection, therefore it is possible that the infection source could be located in Albania. However, molecular epidemiology of HEV in Albania is substantially lacking, therefore this hypothesis could not be demonstrated on a molecular basis. Our study has some limitations; in particular, most recent sequences included in the analysis are referred to cases occurred in a restricted Italian region (Lazio, Central Italy); in addition a limited number of sequences was available before 2016. However, independent from the yearly number of available sequences, the HEV-3 subtype frequency obtained from the whole set of human and animal Italian sequences did not seem to show gross fluctuations over time (not shown).

Overall, the results from this study provide for the first time a direct comparison of HEV-3 subtype distribution in humans and animals in a region that is experiencing a steady increase of incidence of this zoonotic infection. Despite the above mentioned study limitations, it may pioneer a more circumstantiated and robust exploration of the dynamics involved in HEV transmissibility at the human-animal interface, based on a larger availability of shared HEV sequences.

## Data Availability Statement

The datasets generated for this study can be found in NCBI, acccession numbers: MN546866, MN546867, MN509469, MN509470, MN509471, MN444846, MN444845, MN444847, MN444844, MN444839, MN444842, MN444838, MN444840, MN444843, MN444841, MN432489, MN444828, MN444829, MN444830, MN444831, MN444832, MN444833, MN444834, MN444835, MN444836, MN444837, MN444853, MN444852, MN444848, MN444852, MN444849, and MN444850.

## Ethics Statement

The studies involving human participants were reviewed and approved by the Istituto Nazionale per le Malattie Infettive (INMI) Ethical Committee. The patients/participants provided their written informed consent to participate in this study.

## Author Contributions

AG and MC designed the research. MC supervised the study. AG and DL performed the sequences of human strains and analyzed the data. ID and LD performed the sequences of animal strains and phylogenetic analyses. ID wrote the manuscript together with LD and AG. All authors revised the manuscript and approved the final version for submission.

## Conflict of Interest

The authors declare that the research was conducted in the absence of any commercial or financial relationships that could be construed as a potential conflict of interest.

## Footnotes

1 http://blast.ncbi.nlm.nih.gov/

2 https://www.megasoftware.net/

3 https://www.rivm.nl/mpf/typingtool/hev/

4 https://pork.ahdb.org.uk/exports/export-markets

5 https://pork.ahdb.org.uk/prices-stats/news/2018/april/italian-pork-imports-stable/

6 https://ec.europa.eu/eurostat/data/browse-statistics-by-theme
